# Critical perspectives on implementation of evidence-based practice in occupational therapy – Exemplified by Lifestyle Redesign® in a Danish context

**DOI:** 10.1177/03080226211011401

**Published:** 2021-05-11

**Authors:** Stinne Glasdam, Jeppe Oute, Sigrid Stjernswärd

**Affiliations:** 1 Integrative Health Research, Faculty of Medicine, Department of Health Sciences, 5193 Lund University, Lund, Sweden; 2 Faculty of Health and Social Sciences, Department of Health, Social and Welfare Studies, 177041 Campus Drammen, Drammen, Norway; 3 Health-promoting Complex Interventions, Faculty of Medicine, Department of Health Sciences, 5193 Lund University, Lund, Sweden

**Keywords:** Implementation, evidence-based practice, occupational therapy, case study, Lifestyle Redesign®

## Abstract

**Introduction:**

Evidence-based practice is an increasing demand in occupational therapy (OT), although multiple barriers can hinder the translation of research knowledge into practice. The article illuminates the transformation of results from a randomised controlled trial into a practice development project with future practice implementation in mind.

**Method:**

A case study was carried out, consisting of a comparison of the US randomised controlled trials (RCTs) Lifestyle Redesign® and the derived Danish practice development project.

**Results:**

The study showed how results from RCTs of Lifestyle Redesign® were transformed into a practice development project with intentions to implement the programme in a Danish context. The modifications of the US RCT into a practice development project in Denmark compromised the study’s scientific execution. The practice development project was used to legitimise the intervention within OT locally by testing an evidence-based intervention, without using associated scientific tools and without considering barriers and facilitators for implementing the project in clinical practice.

**Conclusion:**

Research design compromises in practice development projects may have implications for the internal and external dynamics of professionalisation processes regarding OT and the recognition of OT as a scientific discipline and an autonomous profession, nationally and internationally.

## Introduction

Historically, occupational therapy (OT) is a relatively young, emerging profession worldwide. Occupational therapy and related basic theory emerged in the United States in the 60–70s and onward (Pierce, 2014). In the United Kingdom, OT became an education at bachelor level in the mid-1990 and at master level in 1999 (Alsop, 2006; Royal College of Occupational Therapists, 1992). In Denmark, the bachelor level in OT was introduced in 2001 (Minister of Education, 2001) and the master level in 2014 (Kjærgaard, 2013). A basic idea in the development of OT practice and research was that the growth of human beings and their well-being are socially conditioned by meaningful activities and that human beings possess potentials that can be achieved through engagement in occupation (Hooper, 2006; Pierce, 2014; Rudman, 2018). However, the profession of medicine and physicians’ practices have also influenced OT, in particular through positivistic thinking and reductionist-oriented practices (Hooper, 2006; Rudman, 2018; Yerxa, 1992). Altogether, there is a range of diverging values related to research tradition within the emerging profession of OT. Yet, research suggests that the ideals of positivism seem to dominate the OT field (Alnervik and Linddahl, 2011) as well as other emerging professions in the field of medicine such as nursing (Glasdam, 2007), physiotherapy (Præstegaard et al., 2015) and maternity care (Benoit et al., 2010). In spite of these tensions, clinicians, researchers and trade unions have a legitimate interest in consolidating and presenting OT as a profession that bases its practice on scientific knowledge in order to enjoy the prestige and legitimacy of a full academic, professional and autonomous profession. In the process of trying to reach this level of political, educational and scientific recognition, the ideal of evidence-based practice, of which the gold standard research design is randomised controlled trials (RCTs), currently plays a pivotal role (Alnervik and Linddahl, 2011; Heiwe et al., 2011; Lindström and Bernhardsson, 2018; Pettersson and Iwarsson, 2017; Upton et al., 2014).

Today, there is a strong research claim for studies using RCTs as design and with focus on clinical care and treatment (Cameron et al., 2005; Von Zweck, 2004; Walshe, 2007). Randomised controlled trials are considered the ‘gold standard’ for evaluating interventions’ efficacy and effectiveness (Streiner and Norman, 2009; Torgerson and Torgerson, 2008). The transition from RCT to implementation research, or so-called knowledge translation of research findings, is a topical theme in political and professional visions on the development of clinical practice and clinical routines (Grimshaw et al., 2012; Wallin, 2009). It is *per se* a transition of a constructed, controlled context to ‘the real reality’ in all its complexity. In a systematic literature review on interventions, it is shown that RCTs often operate as if no contextual impacts exist and, at the same time, the studies consider that the addressed health activity has as its core element the encounter between humans (Glasdam et al., 2015). Not only does the context affect the results that can be achieved but also the people involved will affect what and how things can be done. Through attempts at implementing interventions, potential barriers and facilitators to implementation efforts based on RCT results can be illuminated (Upton et al., 2014). Several studies address these elements through the use of theoretical frameworks (Boström et al., 2012; Rycroft-Malone and Bucknall, 2010; Rycroft-Malone et al., 2004; Wallin, 2009; Ward et al., 2012), facilitating the exploration of contextual factors that need to be addressed to overcome implementation hinders. Such theoretical frameworks help illuminate and map factors on multiple levels, which may interact and affect the implementation process. Such knowledge is valuable to achieve successful implementation of evidence-based knowledge. These models are nevertheless also criticised for having a positivistic ontological and epistemological position, which does not take the complexity of the clinical practice into consideration (Marchal et al., 2013; Pawson et al., 2005; Walshe, 2007). Damschroder et al. (2009) argue that the implementation of an intervention is a social process that is intertwined with the context in which it takes place. Therefore, it is not possible to deliver the same ‘package’ to all participants in an intervention even though this is often assumed in RCTs. It seems important to understand, but also to critically analyse the interaction between people that are challenged with evidence-based knowledge through an implementation. Furthermore, the methods used to gain knowledge about promising practices in OT need to be discussed.

Occupational therapists are committed to lifelong learning, and, over time, the pressure to utilise and implement evidence-based practice has increased (Cameron et al., 2005; Walshe, 2007). Nonetheless, barriers may prevent the implementation of evidence-based knowledge in practice (Cameron et al., 2005). The Californian Lifestyle Redesign® is a good example of an evidence-based programme that reflects a value struggle in OT. Lifestyle Redesign® consists of short intensive interdisciplinary assessments, treatments and activities. The intervention programme is trademarked, which has branding purposes, and it is not suited to protect the content or substance of instruments or interventions (Freres and Finkelman, 2014). A trademark is an instrument of sales promotion and consumer information (Ai Law, 2020). It is only valid in the countries where it is registered. This means that Lifestyle Redesign trademark registered in the United States does not apply in, for example, Denmark. The Lifestyle Redesign® intervention idea builds on the pedagogical ideal of ‘lifelong learning’ for all humans and the ability to relearn day-to-day skills needed to encourage self-confidence, support independence and to promote so-called healthy living among elderly people. The original Lifestyle Redesign® is a manualised OT programme that entails a number of treatment modules, delivered through monthly individual sessions and weekly group sessions, to promote patient education and behaviour changes in daily routines (Clark, 2015; Clark et al., 1997; Jackson et al., 1998). More details about the thematic modules can be found in Table 1 and Supplementary Material file. The programme’s methods rely on didactic presentation, direct experience and exploration and peer exchange. The treatment focuses on patient education, occupational self-analysis, problem-solving, motivational work and implementation of health promoting behaviours (Clark, 2015).

**Table 1.** Descriptions of the studies of Lifestyle Redesign® (United States) and Lifestyle Redesign© (Denmark), inspired by CONSORT 2010 (Schulz et al., 2010) (for further details, see Supplementary Material file).

Content, inspired by CONSORT 2010 (Schulz et al., 2010)	Lifestyle Redesign® (United States)	Lifestyle Redesign® (Denmark)
**Objectives** Specific objectives or hypotheses	To evaluate the effectiveness (Clark et al., 1997) and cost-effectiveness (Hay et al., 2002) of preventive OT services specifically tailored for multi-ethnic, independent living older adults (Clark et al., 1997), including a 6-month follow-up (Clark et al., 2001)	To investigate the effects of Lifestyle Redesign^®^ on older people in Aalborg Municipality, and requirements (economy and other resources) to introduce the programme as a permanent offer in the municipality (Løkken and Overgaard, 2009; Overgaard et al., 2010; Overgaard and Løkken, 2014
**Methods** Trial design including allocation ratio	RCT with 2 cohorts, 16-month time interval and random assignment to 3 arms (Clark et al., 1997; 2001; Jackson et al., 1998). Six-month follow-up (Clark et al., 2001) and 15-month cost-effectiveness analysis of cohort II (Hay et al., 2002)	A single intervention inspired by the US preventive OT programme (Løkken and Overgaard, 2009; Overgaard et al., 2010; Overgaard and Løkken, 2014)
Eligibility criteria for participants	Inclusion criteria: urban, multi-ethnic, independent living, culturally diverse persons, aged ≥60 years, with capacity to benefit in multiple outcome areas from involvement with OT. Exclusion criteria: unable to live independently and marked symptoms of dementia (Clark et al., 1997; 2001; Jackson et al., 1998)	Inclusion criteria: non-employed persons >60 years; social housing resident; chronic disease influenced daily activities; self-reliant or limited need for physical assistance; motivated to work with habits, activities and activity patterns can be part of a group (Løkken and Overgaard, 2009). Exclusion criteria: ongoing psychiatric disorders, dementia and other cognitive disorders (Løkken and Overgaard, 2009)
Settings and locations where the data were collected	Residents and users of senior citizen facilities of two government subsidised apartment complexes for independent living older adults in the Los Angeles area (United States) between 1994 and 1997 (Clark et al., 1997; 2001; Hay et al., 2002; Jackson et al., 1998)	Specific social housing in Aalborg municipality, Denmark in 2007–2009 (Løkken and Overgaard, 2009)
The interventions for each group with sufficient details to allow replication, including how and when they were actually administered	Three intervention arms: 1. OT programme with 2 hours/week of group-based intervention and 1 hour/month of one-on-one therapist–client interaction. Nine modular programmatic units centred on topical content areas. Methods of programme delivery: didactic presentation, peer exchange, direct experience and personal exploration. Four occupational therapists administered the intervention in arm 1, with each therapist overseeing three to four groups of 8–10 elders each (Clark et al., 1997). Arm 2: non-professionally led social activities with diversional group activities, for examplee, viewing films, and playing games. Total number of treatment hours in arm 2 identical to arm 1 except for one-to-one interaction (Clark et al., 1997; 2001; Jackson et al., 1998)). Arm 3: No intervention (Clark et al., 1997; 2001; Hay et al., 2002; Jackson et al., 1998)	Only one intervention group, no other arms. The American, Norwegian and Danish guidelines for ‘Lifestyle Redesign®’ guided the project, called ‘Create your life’ (Løkken and Overgaard, 2009; Overgaard et al., 2010; Overgaard and Løkken, 2014). Weekly group sessions of 2 hours +9 hours of individual intervention over a 9-month period (Løkken and Overgaard, 2009; Overgaard et al., 2010; Overgaard and Løkken, 2014). Seven modular programmatic units centred on topical content areas. Methods of programme delivery: didactic presentation, peer exchange, direct experience and personal exploration (Løkken and Overgaard, 2009). Additionally: individual goals and individual follow-up (9 hours) during intervention + folder with activity summaries related to the intervention, homework, articles, booklets, etc. (Løkken and Overgaard, 2009)
Completely defined pre-specified primary and secondary outcome measures, including how and when they were assessed	Pre-study, a general medical history, physical examination and health status evaluation, including a battery of measurement tools pre-/post-test evaluations (Clark et al., 1997), including 6-month follow-up (Jackson et al., 1998), using a battery of measurement tools (Clark et al., 1997; 2001; Jackson et al., 1998). Cost-effectiveness study: programme costs/person minus healthcare costs (Hay et al., 2002)	Pre, post (4 month) + 8-month follow-up assessments with RAND SF-36 and Canadian Occupational Performance Measure (COPM). Self-constructed semi-structured evaluation interviews immediately post intervention + follow-up (Løkken and Overgaard, 2009; Overgaard et al., 2010). Costs were calculated based on the project leaders' expenses in form of salary and running costs (Løkken and Overgaard, 2009)
How sample size was determined	Power calculation based on RAND SF-36 (Clark et al., 1997). Follow-up (Clark et al., 2001): 79% of the original 361 participants were evaluated both post-test and at the 6-month follow-up. Cost-effectiveness study (Hay et al., 2002): only cohort II included	The project leaders’ preferences. No power calculation (Løkken and Overgaard, 2009)
Generation of random allocation sequence	Computer-generated random numbers and a blocking factor of 6 (Clark et al., 1997)	No
Who generated the random allocation sequence, who enrolled and assigned participants to interventions	Computer-generated random allocation (Clark et al., 1997). Project staff enrolled participants in cooperation with agency managers (Clark et al., 1997). Recruitment of participants through multiple channels (Clark et al., 1997). Only cohort II participants included in the cost-effectiveness study (Hay et al., 2002)	No randomisation. Project leaders enrolled participants. Recruitment through distribution of 500 leaflets to residents’ post-boxes, 19 persons (one man/18 women) showed interest and 8 persons withdrew pre-study. Individual interviews through telephone and home visits to finally include participants. 1 person excluded due to group size (maximum 10 persons), motivation not stated (Løkken and Overgaard, 2009)
Description of the similarity of interventions	Short description of cultural adaptation to Mandarin-speaking subjects (Hay et al., 2002). Detailed description of original intervention (Clark et al., 1997; 2001; Hay et al., 2002; Jackson et al., 1998)	Original concept with some modifications (Løkken and Overgaard, 2009)
Statistical methods used to compare groups for primary and secondary outcomes	Interventions: descriptive and comparative statistical analyses (Clark et al., 1997). Follow-up: descriptive and comparative statistical analyses (Clark et al., 2001). Cost-effectiveness study: descriptive and comparative statistical analyses (Hay et al., 2002)	Individual scores for 5 participants’ RAND SF-36 and COPM assessments, no statistical analysis (Løkken and Overgaard, 2009)
Additional analyses	Intent-to-treat analysis (Clark et al., 1997)	Analysis of evaluation interviews with quotations (Løkken and Overgaard, 2009)
**Results** For each group, the numbers of participants who were randomly assigned, received intended treatment and were analysed for the primary outcome	12 withdrew pre-randomisation. 361 (97%) were randomised (143 in cohort I and 218 in cohort II) to OT group (122), social group (120) and no intervention (119). Treated in three respective arms: 122, 120 and 119. Follow-up: 122, 120 and 119. Withdrew: 20, 20 and 15. Completed: 102,100 and 104 (Clark et al., 1997). Follow-up (Clark et al., 2001): 285 participants evaluated at 6-month follow-up. Cost-effectiveness study (Hay et al., 2002): 163 across the 3 arms in cohort II participated in the survey and costs calculations	19 persons wanted to participate, 8 withdrew pre-study and one excluded due to the maximum group size (10 persons). 10 persons were included in one group. One person withdrew (severe illness) pre-intervention, 4 withdrew during the intervention and 5 persons completed the intervention (Løkken and Overgaard, 2009)
For each group, losses and exclusions after randomisation, together with reasons	Withdrawal at 9 months: arm 1 16%, arm 2 17% and arm 3 12% (Clark et al., 1997). Reasons for withdrawal: death, illness, relocation, personal matters and loss to follow-up. Withdrawal in total: 15%. 21% had dropped out by the 6-month follow-up. Of cohort II (*n* = 218), drop out = 55 due to death/serious illness, change of address, refusal, language barrier and personal reasons, with similar dropout rates across all groups (OT/combined control groups) (Clark et al., 2001)	One participant withdrew pre-intervention due to illness. Four withdrew after five–six month because of illness (own or spouse), relapse of former depression or group conflicts (Løkken and Overgaard, 2009)
Periods of recruitment and follow-up	1994–1996 (Clark et al., 2001)	January 2008–May 2009 (Løkken and Overgaard, 2009)
Baseline demographic and clinical characteristics for each group	No significant differences in demographic characteristics across groups. 361 non-English speaking men (35%) and women (65%), mean age of 74.4 years, residing in or using government-subsidised apartment complexes for independent seniors and high risk of poor health associated with low socio-economic status (Clark et al., 1997; Jackson et al., 1998). Follow-up (Clark et al., 2001): 79% (*n*=285) of original sample, with mainly similar demographic characteristics. Cost-effectiveness study (hay t al., 2002): cohort II with same baseline characteristics as cohort I	One group of nine persons: all were independent living and self-reliant; 7 lived alone, 1 with a spouse and 1 with a child; all reported a form of functional reduction, aged 60–68 years; all Danish women, 7 with children; different kinds of chronic diseases and 4 active in other social groups (Løkken and Overgaard, 2009)
For each primary and secondary outcome, results for each group, and the estimated effect size and its precision	Short- and long-term benefits attributable to OT treatment were measured and described. No differences were found in the two control groups (Clark et al., 1997). Overall, healthcare costs were lower for the OT group than the 2 control groups (Hay et al., 2002)	COPM calculated and RAND SF-36 described for the 5 participants, showing progress during intervention and status quo or decline at follow-up. No comparative or significance analyses due to the limited sample size. The interview evaluation described Lifestyle Redesign® as a useful and effective concept for elderly people (Løkken and Overgaard, 2009)
**Discussion** Trial limitations	Limited generalisability due to sample characteristics (Clark et al., 1997; 2001), differing treatment administration approaches (Clark et al., 2001), limited follow-up interval (Clark et al., 2001; Hay et al., 2002), single cohort inclusion, potential confounding factors and follow-up without continued treatment (Hay et al., 2002)	No trial limitations or potential sources of bias were discussed (Løkken and Overgaard, 2009)
Generalisability (external validity and applicability) of the trial findings	The results may not be generalised to older adults in different living situations, of different socio-economic status or in diverse contexts (Clark et al., 1997; 2001). Limitations pertaining to generalisability of cost-effectiveness results (Hay et al., 2002)	The authors suggested implementation in Danish municipalities, with consideration taken to participants' pre-RAND SF-36 scores, cognitive and social level of functioning (Løkken and Overgaard, 2009)
Conclusion	OT, individual adaptation of programme to elders’ needs and contextual programme modifications, professional leadership and judgement regarded as key ingredients in enabling elders to benefit from activity (Jackson et al., 1998). Significant benefits for the OT preventive treatment group across various health, function and quality-of-life domains. Preventive OT health programmes may mitigate against the health risks of older adulthood (Clark et al., 1997), with retained benefits at long-term follow-up (Clark et al., 2001). Preventive OT demonstrated cost-effectiveness and a decreased medical expenditures trend (Hay et al., 2002)	The report authors concluded that: - the Lifestyle Redesign® programme was useful and effective for the elderly in Aalborg municipality. - occupational therapists must be theoretically and practically schooled in the programme have access to OT professional supervision (Løkken and Overgaard, 2009)

Note: OT: occupational therapy.

The programme puts forward evidence-based recommendations for OT interventions that aim to improve health and wellness by preventing and managing chronic conditions by building healthier lifestyles (Clark, 2015; Clark et al., 1997; Jackson et al., 1998). Lifestyle Redesign® is launched internationally, partly to respond cost-effectively to the so-called elderly wave, which politically has been deemed economically burdensome for society (Iversen and Kjellberg, 2019). Lifestyle Redesign® is an acknowledged programme in the Western world as its applicability, design and effects are founded on a forerunning RCT of Lifestyle Redesign® in California. In some countries, the RCT is repeated in modified forms, for example, the United States (Colorado), Sweden, Norway and Denmark (Cassidy et al., 2017; Johansson, 2009; Johansson and Björklund, 2016; Kragbæk, 2008; Lund et al., 2012; Løkken and Overgaard, 2009; Svendsen and Lillebø, 2007; Svendsen and Otterholt, 2006). In other countries, the programme is transformed into guidelines, for example, the United States (New York and California), Canada and the United Kingdom (Clark, 2015; Horowitz and Chang, 2004; Juang et al., 2018; Levasseur et al., 2019; Mountain et al., 2008; USC Mrs, 2020). Previous studies show that Lifestyle Redesign® has been tested in different cultural contexts and in varied populations with diverse conditions, such as stroke (Lund et al., 2012; Ng et al., 2013), diabetes (Pyatak et al., 2019) and chronic pain (Simon and Collins, 2017). Examples of cultural adaptations can be seen (e.g. Levasseur et al., 2019; Matuska et al., 2003). Adaptations may be necessary for the intervention to fit especially targeted populations that may differ from those in prior studies, for example, in terms of sociodemographic and health characteristics and thus needs and abilities (Levasseur et al., 2019). The political-clinical emphasis on cost-effective, evidence-based interventions in elderly care stems from a broader attempt to set the standard for new health policy and routine practice across care settings in public welfare systems. Those standards rely on RCTs (Glasdam, 2007). With Lifestyle Redesign® in a Danish context serving as a case, this article aims to illuminate the transformation of the results of RCT into practice development project with future practice implementation in mind.

## Materials and methods

The study was based on a case study, consisting of a comparison of the RCT Lifestyle Redesign® from the United States and the derived Danish practice development project, with an analysis inspired by the CONSORT 2010 checklist for RCT (Schulz et al., 2010). The analysis was based on written, publicly available articles and reports that describe the original RCT in the United States and the derived Danish practice development project.

### Strategy for collecting empirical material

In a previous study exploring Danish practice development projects, the Danish Lifestyle Redesign® project was part of the empirical material (Glasdam and Munksgaard, 2016; Glasdam and Sudmann, 2021). Descriptions of the practice development project Lifestyle Redesign® in a Danish context were first searched through database searches in PubMed and CINAHL, using the keywords: Lifestyle Redesign and Denmark. No literature was found. Second, a free search on municipal and trade union websites and the web search engine Google were made. We found three texts about the project: a report of the practice development project (Løkken and Overgaard, 2009), a news flash and a popular scientific article about the project, all published on the Danish Association of Occupational Therapists’ webpage (Overgaard et al., 2010; Overgaard and Løkken, 2014).

We searched manually for articles about the initial RCT of Lifestyle Redesign® in California, the United States, through database searches in PubMed and CINAHL, using the keywords: Lifestyle Redesign® and California. We also searched relevant articles’ reference lists manually. These searches resulted in four scientific articles about the RCT on Lifestyle Redesign® in California (Clark et al., 1997; 2001; Hay et al., 2002; Jackson et al., 1998).

### Analytical strategy

The checklist CONSORT 2010 (Schulz et al., 2010) functioned as inspiration for the construction of analytical questions to compare the original RCT conducted in California in the late 1990s and the derived practice development project conducted in Denmark in 2007–2009. Lifestyle Redesign® (US) is analysed through the four scientific articles (Clark et al., 1997; 2001; Hay et al., 2002; Jackson et al., 1998), which are handled as a single unit of text that describes the primary US project in its entirety. Lifestyle Redesign® (Denmark) is analysed through the three texts (Løkken and Overgaard, 2009; Overgaard et al., 2010; Overgaard and Løkken, 2014), which were also regarded as a single unit of text describing the Danish project in its entirety. First, the analytical questions were inserted into a matrix together with the actual analysis of Lifestyle Redesign® from the United States and Denmark, respectively. Second, the modifications from the US RCT to the Danish practice development project were analysed.

## Results

Descriptions of the studies of Lifestyle Redesign® (the United States) and Lifestyle Redesign® (Denmark), inspired by CONSORT 2010 (Schulz et al., 2010), were presented in Table 1 and Supplementary Material file.

As seen in Table 1, the main modifications in the practice development project in Denmark pertain to the study’s scientific design, with multiple deviations from the original RCT design. Differences were noted in terms of sample characteristics and size, study design, follow-up and, of course, context as the point of the Danish study was to test the programme in a new context. The central aim of the studies was similar (effectiveness studies), although the US studies had additional aims (effectiveness study including follow-up and cost-effectiveness study). The programme itself was (mostly) the same in terms of format and contents, with some modifications in the Danish programme.

Overall, the modifications of the original RCT from the United States to the Danish practice development project were simplifications. The design was reduced from a RCT with three arms (preventive OT programme, non-professionally led social activities and no intervention) to ‘testing’ of preventive OT programme in one sample and thus only one arm and no randomisation. The number of participants was drastically reduced. The US sample had 361 participants (2 cohorts), out of which 122 (102 completed) were in the intervention arm and divided into groups of 8–10 persons. There was only one group of nine persons (five completed) in the Danish study. The United States recruited participants strictly adhered to predefined inclusion and exclusion criteria. The participants were generally identified as in high risk of poor health due to low socio-economic status. The Danish study also had predefined inclusion and exclusion criteria, but it did not include socio-economic status. These criteria were not used strictly. Primarily, they were used to screen possible participants. Secondly, participants were chosen through subjective assessments of their ability to fit in the intervention, with a maximum set at totally 10 persons. Further, the assessment and evaluation design of the intervention was modified. In the US RCT, there was a range of measuring tools and statistical descriptive and comparative analyses within and across group(s). In the Danish study, only SF-36 and COPM were used as assessment tools in combination with individual interviews. The measurements were presented for each participant, with no comparative analyses across group participants. The US RCT was transparent and strictly conducted according to scientific tradition. It included a follow-up study at 6 months and a cost-effectiveness study. Generalisation and limitations were discussed in the US studies. Opposite, the small sample, recruitment procedure and intervention assessment in the Danish study/project prevent generalisation, although the authors did not address these points but rather suggest implementation of the programme within Danish municipalities. Further, the Danish study did not consider participants’ socio-economic status, even though the US RCT showed that generalisation to older adults with different living situations, socioeconomic status or contexts was not possible. A rich description of the sample’s characteristics nevertheless permitted readers to reflect about transferability of the Danish results, bearing in mind that the research design’s weaknesses make the results scientifically weak. All in all, the Danish study’s conclusions were not consistent with its results as the authors did not balance strengths and weaknesses in the project and its design, nor considered potential effects of, for example, confounding factors. What comes through as a noteworthy observation in the Danish project were the conclusions pertaining to the programme’s effectiveness, which were based on a (weak) study design, and included recommendations to implement the programme within regular OT services in Denmark. There was a lack of critical discussion of the project’s limitations.

## Discussion

The current study illuminated similarities and differences in how Lifestyle Redesign® was tested and assessed in its original context and format in the United States and in a practice development project in Denmark, based on empirical data that were available online. As a practice development project, the Danish study is of interest. It can be seen as a first attempt to implement an American intervention on Danish soil. The discussion will focus on potential consequences of the transformation of the Lifestyle Redesign® intervention programme into a practice development project with future practice implementation in mind in relation to OT as a scientific and academic discipline and practice.

Based on a subjective evaluation by only five participants and two project leaders, the practice development project concludes that the project is good, effective, health preventive and that it is worth investing and implementing as an intervention by occupational therapists in a Danish context hereafter. And this despite the Danish occupational therapist Kragbæk’s (2008), critical discussion that one should be careful about making a direct comparison with the US study Lifestyle Redesign® in studies with few participants and in an adapted form. Kragbæk (2008) further states that there are significant different sociocultural conditions in Denmark compared to the United States. This conclusion may be viewed in relation to the fact that OT is a relatively new academic discipline in Denmark (Kjærgaard, 2013) with a need to prove its academic worth, which may help explain that both trade unions and professional colleges support a legitimisation of the development project. However, the shift from a RCT in the United States to a practice development project in Denmark implies that the study’s scientific basis dissipates. Practice development projects are in a grey zone between scientific study and clinical practice. This means that they become neither/nor projects that most of all set themselves up as political and professional visions and dreams (Durkheim, 1956). It claims to test a scientific study in a concrete, self-defined practice, without using the scientific tools associated with this thinking, and at the same time, subjective assessments functioned as the most important evaluation parameters for whether the project is successful and recommendable for implementation in a Danish context going forward. The case shows how a practice development project testing Lifestyle Redesign® in a new cultural context risks, consciously or not, compromising the project’s original scientific execution and hence also the intervention’s value, through modifications in the testing design. Based on what can be deemed as weak scientific evidence, the implementation of Lifestyle Redesign® was nonetheless legitimated within OT services in the new context, that is, Denmark. Examples of deviations from the original RCT design are the lack of control arms, modified inclusion and exclusion criteria. Deviations from original designs and intervention contents need to be problematised and discussed. As far as the authors could see from the current study’s empirical data, none of these circumstances were reflected upon in the conclusion of the practice development project. The practice development project moves from a scientific context with carefully defined rules for how an RCT is performed towards a clinical and political context, where the ability to legitimise a project and convince decision makers about implementation is important.

When transferring an intervention from one context, one population to another, adaptations may be necessary (Levasseur et al., 2019). This includes cultural and language adaptations as seen in the current practice development project in Denmark, but also tailorising the intervention to successfully address the targeted population’s specific needs and prerequisites (Kielhofner et al., 2004; Levasseur et al., 2019). However, the Danish project did not motivate or problematise the reduced number of outcomes for the intervention for that specific population, which is important whether using RCTs or other research designs to assess the intervention’s effectiveness and value for its participants (Kielhofner et al., 2004; Simon and Collins, 2017). Evidence-based knowledge needs to be translated into practice in a way that fits the context in question. The Danish development project was inspired by the American and Norwegian projects, focussing a number of thematic modules deemed as relevant for the participants. The intervention’s methods were largely similar to the original intervention. However, barriers and facilitators that can affect the implementation process also need to be mapped and addressed for a successful implementation process. Without knowledge of barriers and facilitators, implementation efforts may be thwarted. Pyatak et al., 2019, for instance, carried out a combined effectiveness–implementation study of Lifestyle Redesign® for patients with diabetes. Some of the implementation steps addressed in their project included anchoring the idea with key stakeholders, securing funds, educating providers and clinic staff and providing maintenance and support in delivering the intervention (Pyatak et al., 2019). A sense of ownership of the project and the use of champions may also facilitate the implementation process (Proctor et al., 2009; Rycroft-Malone and Bucknall, 2010). In the Danish project, the project group consisted of a few persons that carried out most steps in the project, without anchoring or critically assessing it in collaboration with other affected parties or stakeholders. Besides assessments of the intervention’s feasibility and efficiency, further aspects such as appropriateness, acceptability, fidelity and timeliness of the intervention shall be appraised (Pyatak et al., 2019). In light of the trademarked Lifestyle Redesign® in the United States, fidelity assessments may need adjustments adapted to the intervention’s specific customisations into the non-trademarked registered version of the Danish version of Lifestyle Redesign®. The accumulation of evidence about outcomes and process may thence help enhance the services in a dynamic process (Kielhofner et al., 2004).

The Danish approach to the practice development project might have implications for both the internal and external dynamics of professionalisation and recognition of OT as an academic, scientific discipline, internally among occupational therapists nationally and internationally, and externally in the medical field and society in general (Cooper, 2012). Markey (2014) argues that the ideal notions of professionalism differ from other ways of organising the control of work, namely consumerism, which celebrates competition and cost, and managerialism, which celebrates efficiency through standardisation and the minimisation of discretion. Lifestyle Redesign®, assessed through RCTs in California or put forward as a practice development project in Denmark, can be an important driver or catalyst for academisation and professionalisation of OT. However, the Danish choice of methods has unintended consequences. The professionalisation efforts represent a serious attempt at legitimising a new form of practice and institutionalised professionalism, which claims to be evidence-based and wants to guide current practice for occupational therapists. At the same time, professionalisation can also be seen as a strategy for maintaining and expanding clinical tasks and functions for occupational therapists (Bresnen, 2013) by separating OT from other professional groups in the medical field. However, the trade union has a legitimate interest in consolidating its representatives and their work area in the medical field. In Denmark, the occupational trade union supports the idea of Lifestyle Redesign® by publishing articles, manuals and notes in their journals and on their webpages about the above described practice development project (Kragbæk, 2008; Løkken and Overgaard, 2009; Overgaard et al., 2010; Overgaard and Løkken, 2014). In addition, the Danish Health Authority (Niclasen et al., 2017) supports the idea of Lifestyle Redesign®. Today, Lifestyle Redesign® has been initiated in several practice development projects, based on the first Danish practice development project (Berthelsen et al., 2016; Johannessen, 2012). The same went for teaching offerings in both University Colleges and private auspices (Christensen, 2020; UC Copenhagen, 2020) and as research focus in bachelor projects (Jepsen, 2010; Svane and Thyrrestrup, 2010). If the academisation and professionalisation of OT are to be taken seriously, it also calls for a trade union’s critical assessment of their articulations of evidence-based knowledge. Kragbæk (2008) questions whether there is any need in Denmark for health-promoting and preventive programmes in the form of Lifestyle Redesign®, and in continuation of this, we can consider whether such a project under Danish auspices is important for the target group or for occupational therapists’ research merits. The Danish development project can be considered a pre-academic work, driven by enthusiastic, academic novices with the best intentions for the project participants and for OT. At first glance, it seems important to repeat the project in a Danish context, with a rigorous scientific methodological design, and put such an endeavour in relation to whether the programme should be further implemented in clinical practice or discouraged. Unmotivated claims and conclusions that are based on what can be deemed scientifically weak evidence risk having the opposite effect on scientific credibility and efforts at academisation and professionalisation of OT.

## Methodological considerations/limitations

The current study is a case study that can illuminate and explain the single unit for understanding a larger class of (similar) units (Flyvbjerg, 2006). The study is based on scientific articles, reports and publicly available material, that is, secondary data and not primary data. This is a limitation per se; nonetheless, the current study shows how such data can be made the basis for national recommendations. As discussed in the current article, such material and how it is handled can have implications for academisation and professionalisation of OT, which are the focus of the current article. A temporality factor needs to be considered as the named reports and scientific publications were published at a time when OT was not yet offered as an education at master level in Denmark. The analysis was inspired by the CONSORT checklist (Schulz et al., 2010), with minor deviations. Some questions were not included as they were not deemed essential for the current study’s aim. The authors were hence not fully compliant to the original formula but do not estimate that these deviations notably affect the study’s results. The researchers analysed data separately then co-jointly discussed their findings and reached consensus, strengthening the findings’ reliability.

## Conclusion

The article showed how a transition of a RCT into a practice development project with future practice implementation in mind risks to end up in a grey zone, representing neither intervention or implementation research, nor clinical practice. Lifestyle Redesign® as a case demonstrated how modifications and simplifications of a US RCT into a Danish practice development project were problematic and compromised the scientific execution. The Danish study’s conclusions were not consistent with its results as the authors did not balance strengths and weaknesses in the project and its design nor consider limitations of the project. The US RCT and derived projects might have implications for internal and external dynamics of professionalisation processes regarding OT and the recognition of OT as a scientific discipline and an autonomous profession, nationally and internationally. The article calls for further research in two directions. On the one hand, it calls for a new RCT of Lifestyle Redesign® in a Danish context or at least a study with a sound research design. It further calls for an implementation study, based on a version of the intervention that is systematically customised for the targeted population in Denmark, and with focus on facilitators and barriers to implementation. On the other hand, it calls for studies of professionalisation and academisation of OT from sociological perspectives to understand the complexities and consequences of those processes.

## Key findings

- Compromises in scientific design can undermine professionalisation and academisation efforts in OT.
- Lack of adherence to scientific research standards may jeopardise implementation efforts of evidence-based practice.

## What the study has added

Enthusiasm and good intentions need support of scientific rigour to build internal and external professional acknowledgement of OT in the medical field and academia, promoting professionalisation and academisation of OT.

## Supplemental Material

sj-pdf-1-bjo-10.1177_03080226211011401 – Supplemental Material for Critical perspectives on implementation of evidence-based practice in occupational therapy – Exemplified by Lifestyle Redesign® in a Danish context Supplemental Material, sj-pdf-1-bjo-10.1177_03080226211011401 for Critical perspectives on implementation of evidence-based practice in occupational therapy – Exemplified by Lifestyle Redesign® in a Danish context by Stinne Glasdam, Jeppe Oute and Sigrid Stjernswärd in British Journal of Occupational Therapy
