# Supplementary material for: Critical perspectives on implementation of evidence-based practice in occupational therapy – Exemplified by Lifestyle Redesign® in a Danish context
Source: Br J Occup Ther. 2021 May 11;85(3):208–18. doi: 10.1177/03080226211011401 (PMC12033703; doi:10.1177/03080226211011401)
Supplement: sj-pdf-1-bjo-10.1177_03080226211011401 – Supplemental Material for Critical perspectives on implementation of evidence-based practice in occupational therapy – Exemplified by Lifestyle Redesign® in a Danish context [file sj-pdf-1-bjo-10.1177_03080226211011401.pdf]

Supplementary file:

Detailed descriptions of the studies of Lifestyle Redesign® (US) and Lifestyle Redesign© (Denmark), inspired by CONSORT 2010 (Schulz et al., 2010)

**Content, inspired by CONSORT 2010** (Schulz et al., 2010)	**Lifestyle Redesign® (US)**	**Lifestyle Redesign® (Denmark)**
**Objectives**<br><br>Specific objectives or hypotheses	Aims: To evaluate the effectiveness (Clark et al., 1997) and cost-effectiveness (Hay et al., 2002) of preventive OT services specifically tailored for multi-ethnic, independent-living older adults (Clark et al., 1997), including a 6-month follow-up (Clark et al., 2001). Hypotheses: programme participation positively affects physical health, daily functioning and psychological well-being as compared to control groups (Clark et al., 1997), including long-term health improvements (Clark et al., 2001), with reduction of long-term healthcare costs and potential cost-effectiveness (Hay et al., 2002)	To investigate the effects of Lifestyle Redesign® on older people in Aalborg Municipality, and requirements (economy and other resources) to introduce the programme as a permanent offer in the municipality (Løkken and Overgaard, 2009; Overgaard et al, 2010; Overgaard and Løkken, 2014).
**Methods**	RCT with 2 cohorts with a 16- month time-interval and random assignment to three intervention arms for 9	A single intervention inspired by the US preventive occupational therapy program (Løkken and Overgaard,

Description of trial design including allocation ratio	months: 1/3 received preventive OT, 1/3 participated in non-professionally led social activities, and 1/3 got no intervention (Clark et al., 1997; 2001; Jackson et al., 1998). Six-month follow-up (Clark, 2001) and cost-effectiveness analysis of cohort II at 15 month (Hay et al., 2002)	2009; Overgaard et al, 2010; Overgaard and Løkken, 2014).
Eligibility criteria for participants	Inclusion criteria: urban, multi-ethnic, independent-living, culturally diverse persons, aged ≥60, with capacity to benefit in multiple outcome areas from involvement with OT.<br>Exclusion criteria: unable to live independently, marked symptoms of dementia (Clark et al., 1997; 2001; Jackson et al., 1998).	Inclusion criteria:<br>- Non-employed persons > 60 years.<br>- Social housing resident.<br>- Having a chronic disease affecting or threatening to influence daily activities.<br>-Self-reliant or with limited need for physical assistance.<br>- Willing to make an effort for a life with meaningful activities.<br>- Motivated and able to work with own roles, habits, activities and activity patterns, with the aim of promoting own health through changed lifestyles.<br>- Can be part of a group (Løkken and Overgaard, 2009).<br><br>Exclusion criteria: Ongoing psychiatric disorders; dementia; other cognitive disorders (Løkken and

		Overgaard, 2009).
Settings and locations where the data were collected	Residents and users of senior citizen facilities of two government subsidised apartment complexes for independent living older adults in the Los Angeles area (US) between 1994-1997 (Clark et al., 1997; 2001; Hay et al., 2002; Jackson et al., 1998).	Specific social housing in Aalborg municipality, Denmark in 2007-2009 (Løkken and Overgaard, 2009).
The interventions for each group with sufficient details to allow replication, including how and when they were actually administered	Three intervention arms: 1. OT programme with 2 hrs/week of group-based intervention and 1 hr/month of one-on-one therapist-client interaction. Modular programmatic units centred on topical content areas such as 1) Introduction to the Power of Occupations; 2) Ageing, Health, and Occupation; 3)Transportation; 4) Safety; 5) Social Relationships; 6) Cultural Awareness; 7) Nutrition; 8) Finances and 9) Integrative Summary. Methods of programme delivery: Didactic Presentation, Peer Exchange, Direct Experience and Personal Exploration. Four occupational therapists administered the intervention arm of the project, with each therapist overseeing three to four groups of 8-10	Only one intervention group, no other arms. The American, Norwegian and Danish guidelines for "Lifestyle Redesign®" guided the project, called 'Create your life' (Løkken and Overgaard, 2009; Overgaard et al, 2010; Overgaard and Løkken, 2014). Weekly group sessions of 2 hours + 9 hours of individual intervention over a 9-month period (Løkken and Overgaard, 2009; Overgaard et al, 2010; Overgaard and Løkken, 2014). Modular programmatic units centred on topical content areas: 1) Activity, ageing and health; 2) Physical health and activity; 3) Safety and activity; 4) Transport and activity; 5) Social relations and activity; 6) Nutrition and activity; and 7) Finances and activity. Methods of programme delivery:

	elders each (Clark et al., 1997). Arm 2. Non-professionally led social activities with diversional group activities, e.g. viewing films, playing games, attending dances, and community outings. Total number of treatment hours in arm 2 identical to arm 1 with 4 sessions/month of 2:15 hours each, but no one-to-one interaction (Clark et al., 1997; 2001; Jackson et al., 1998). Arm 3. No intervention (Clark et al., 1997; 2001; Hay et al., 2002; Jackson et al., 1998).	Didactic Presentation, Peer Exchange, Direct Experience and Personal Exploration (Løkken and Overgaard, 2009). Additionally: individual goals and individual follow-up (9 hrs) during intervention + folder with activity summaries related to the intervention, homework, articles, booklets, etc. (Løkken and Overgaard, 2009).
Completely defined pre-specified primary and secondary outcome measures, including how and when they were assessed	Prior to the 9-month experimental treatment phase, a general medical history, physical examination, and health status evaluation (using the Modified Mini-Mental State Examination, the short form Geriatric Depression Scale, LaRue Global Health Assessment, and Tinetti Balance Examination was performed for each participant.<br>Pre-post test evaluations (Clark et al., 1997), including 6-month follow-up (Jackson et al., 1998), using RAND 36-item Short Form Health Survey (RAND SF-36), Functional Status Questionnaire (FSQ), Life Satisfaction Index-Z (LSI-Z), Center for Epidemiologic	Pre, post (4 month) + 8-month follow-up assessments with RAND SF-36 and Canadian Occupational Performance Measure (COPM). Semi-structured evaluation interviews developed for the project immediately post intervention + at 8-month follow-up (Løkken and Overgaard, 2009; Overgaard et al, 2010). Costs were calculated based on the project leaders' expenses in form of salary and running costs (e.g. supervision, literature, etc.) (Løkken and Overgaard, 2009).

	Studies—Depression Scale (CES-D), and the Medical Outcomes Study (MOS) Health Perception Scale (Clark et al., 1997; 2001). Only participants in Cohort II were evaluated using RAND SF-36 (Clark et al., 2001).<br><br>Cost-effectiveness study (Hay et al., 2002): Program costs/person minus Healthcare costs. Use of healthcare services was determined by telephone interview during and after the treatment phase. A conversion algorithm was applied to the RAND SF-36 to derive a preference-based health-related quality of life index, quality-adjusted life years (QALYs), and the incremental cost-effectiveness ratio for preventive OT relative to the combined control group (Hay et al., 2002).	
How sample size was determined	Power calculation based on an estimated 20% attrition rate over 9 months, with 0.5 level (1-tailed) testing levels (cohort I, projected sample of 360 with a 2:1 allocation ratio) and $\geq$0.4 level for RAND SF-26 (cohort II, projected sample of 220) (Clark et al., 2001).	The project leaders' preferences. No power calculation (Løkken and Overgaard, 2009).

	Follow-up (49): 79% of the original 361 participants in Clark et al (1997) were evaluated both post-test and at the 6-month follow-up.<br><br>Cost-effectiveness study (Hay et al., 2002): Only Cohort II included.	
Method used to generate the random allocation sequence	Computer-generated random numbers and a blocking factor of 6 (Clark et al., 1997).	No.
Who generated the random allocation sequence, who enrolled participants, and who assigned participants to interventions	Computer-generated random allocation, with the researchers allocating subjects in two cohorts to 1 of 3 treatment groups within strata defined by language testing (Clark et al., 1997). Project staff enrolled participants in cooperation with agency managers (Clark et al., 1997). Recruitment of participants through staffed recruitment tables in facility lobbies, at on-site functions (e.g. dances, coffee hours), flyers, residence newsletter articles, presentations at regular meetings (e.g. Senior Citizens Club), letters placed under residents' doors (Clark et al., 1997). Only cohort	No randomization. The project leaders enrolled and assigned participants to the intervention. Recruitment through distribution of 500 leaflets to residents' post-boxes, 19 persons (one man/18 women) contacted the researchers for participation in the project. Eight persons withdrew in the recruitment phase. The project leaders conducted individual interviews, by both telephone and home visits, to finally include participants. One person excluded due to group size (maximum 10 persons), motivation not stated (Løkken and Overgaard, 2009).

	II participants included in the cost-effectiveness study (Hay et al., 2002).	
Description of the similarity of interventions	Short description of cultural adaptation to Mandarin-speaking subjects (Hay et al., 2002). Detailed description of original intervention used across all US studies (Clark et al., 1997; 2001; Hay et al., 2002; Jackson et al., 1998).	Reproduction of the original concept with some modifications: Exclusion of original topical content areas (Introduction to the Power of Occupations; Cultural Awareness; Integrative Summary). Added topical content area (Physical health and activity) (Løkken and Overgaard, 2009).
Statistical methods used to compare groups for primary and secondary outcomes	Scores on measurements, $\chi 2$ analysis and analysis of variance to test for differences at baseline across the 3 arms (Clark et al., 1997). Signed change scores for treatment effects for each outcome variable (Clark et al., 1997). Analysis of variance to determine demographic factors related to change scores independent of treatment group (Clark et al., 1997). Analysis of covariance to test for equivalency between arms 2 and 3 (0.5 level, 2-tailed), and differences between arm 1 and the combined control groups (0.5	Individual scores for 5 participants' RAND SF-36 and COPM assessments (Løkken and Overgaard, 2009).

	level, 1-tailed) (Clark et al., 1997).<br><br>Follow-up (61): For demographic and baseline history and physical examination variable, 2-tailed tests for differences between participants with/without follow-up and between treatment groups (OT vs combined control) for participants with follow-up (Clark et al., 2001). For each outcome variable, evaluations of treatment effects through signed change scores calculations and analysis of covariance to test for change score differences between OT treatment group and combined control group. Regression analyses to impute values for missing scores. Alpha level 0.05, 1-tailed assessments to examine OT outcomes (Clark et al., 2001).<br><br>Cost-effectiveness study (Hay et al., 2002): Analysis of variance methods and analysis of covariance, at 0.5 level (Hay et al., 2002).	
Methods for additional analyses	Intent-to-Treat analysis for subjects who completed the study (Clark et al., 1997)	Analysis of evaluation interviews with quotations based on five participants’ individual courses (Løkken and

		Overgaard, 2009).
**Results**<br><br>For each group, the numbers of participants who were randomly assigned, received intended treatment, and were analysed for the primary outcome	Out of 373 eligible volunteers, 12 withdrew pre-randomization. The 361 volunteers (97%) were randomized (143 in cohort I, 218 in cohort II) to OT group (122), social group (120), no intervention (119). Treated in three respective arms: 122, 120, 119. Followed-up: 122, 120, 119. Withdrew: 20, 20, 15. Completed: 102,100,104 (Clark et al., 1997).<br><br>Follow-up (Clark et al., 2001): 285 participants evaluated at 6-month follow-up. Percentages of participants with follow-up evaluations did not differ between treatment groups (Clark et al., 2001).<br><br>Cost-effectiveness study (Hay et al., 2002): Out of the 218 participants in cohort II, 55 did not complete the survey, dropout rates similar across all groups (OT and combined control group). Answered the survey: OT group 55, combined control group 112. Follow-up data available for 47 out of 51 in OT group, 47 out of 53 in	19 persons wanted to participate, 8 withdrew before final inclusion, one person was excluded due to the maximum group size of 10 persons. 10 persons were included in one group, no randomization. One person withdrew (severe illness) pre-intervention, 4 withdrew during the intervention, 5 persons completed the intervention (Løkken and Overgaard, 2009).

	social activities control group, and 54 out of 59 in no intervention group (Hay et al., 2002). Costs calculations for 51 in OT group, 53 in social activities group, and 59 in no intervention group (Hay et al., 2002).	
For each group, losses and exclusions after randomisation, together with reasons	Withdrawal at 9 months: 20 persons (16%) in the OT group, 20 persons (17%) in the social activity control group, and 15 persons (12%) in the non-treatment control group (Clark et al., 1997). Reasons for withdrawal: death (8), illness (3), relocation (13), personal matters (11), and loss to follow-up (20). Withdrawal in total: 15% (28).<br>21% had dropped out by the 6-month follow-up (49). Of cohort II (n=218), 55 did not complete the medical expenditure survey due to death/serious illness (3), change of address (4), refusal (7), language barrier (17), personal reasons (24), with similar dropout rates across all groups (OT/combined control groups) (Hay et al., 2002).	One out of ten included participants withdrew pre-intervention due to illness. Four withdrew after five-six month because of illness (own or spouse), relapse of former depression, or group conflicts (Løkken and Overgaard, 2009).

Dates defining the periods of recruitment and follow-up	1994-1996, including a 6-month follow-up (Clark et al., 2001).	January 2008 - May 2009 (Løkken and Overgaard, 2009).
Baseline demographic and clinical characteristics for each group	No significant differences in demographic characteristics across groups. 361 non-English speaking African American, Asian, Caucasian, and Hispanic, men (35%) and women (65%), aged 60+ years (mean age =74.4 years), residing in or using government-subsidised apartment complexes for independent seniors, and identified as in high risk of poor health due to low socio-economic status (Clark et al., 1997; Jackson et al., 1998).<br><br>Follow-up (Clark et al., 2001): 79% (n=285) of original sample evaluated at follow-up, with mainly similar demographic characteristics. However dropouts (n=76) had lower scores on Tinetti Balance Examination (p=.04), LaRue Global Health Assessment (p=.05), reported taking more medication (p=.03).<br><br>Cost-effectiveness study (Hay et al., 2002): cohort II	One group of nine persons:<br>- All were independent-living.<br>- All were self-reliant.<br>- 7 lived alone, one with a spouse, one with a child.<br>- All reported a form of functional reduction.<br>- Aged 60-81.<br>- All Danish women, 7 with children.<br>- Different kinds of chronic diseases.<br>- 4 active in other social groups (Løkken and Overgaard, 2009).

	(55 dropouts) with same baseline characteristics as cohort I. Significantly fewer Hispanic and English-speaking Asians completed the survey.	
For each primary and secondary outcome, results for each group, and the estimated effect size and its precision	Benefit attributable to OT treatment found for the quality of interaction scale on the Functional Status Questionnaire (p=.03), Life Satisfaction Index-Z (p=.03), Medical Outcomes Study Health Perception Survey (p=.05). For 7 of 8 scales on the RAND SF-36: bodily pain (p=.03), physical functioning (p=.008), role limitations attributable to health problems (p=.02), vitality (p=.004), social functioning (p=.05), role limitations attributable to emotional problems (p=.05), and general mental health (p=.02). No differences were found in the two control groups (Clark et al., 1997)<br><br>Long-term benefit attributable to preventive OT found for the quality of interaction scale of the Functional Status Questionnaire and for six of eight scales on the RAND SF-36: physical functioning, role functioning, vitality, social functioning, role emotional, and general	COPM calculated for the 5 participants. Four participants showed progress in relation to their own goals at the end of the intervention. At follow-up, four participants showed decline compared to end intervention; of these, however, 2 participants showed progress in relation to the start. No comparative or significance analyses due to the limited sample size (Løkken and Overgaard, 2009). RAND SF-36 showed a 3-18% percentage increase pre-post intervention on all components except Mental Health, which increased by 50%. From end intervention to follow-up: status quo on Social Function and Role Emotional; 0-14% decrease on all other components, expect Role Physical with a 40% decrease. From start to follow: 1-10% improvement in the components Physical function, Social Function, Mental Health and Mental Component Score + 50% progress on the Role Emotional component. From start to follow: 2-8% decrease in the components Bodily pain, General Health Perceptions,

	mental health (p, .05). Approximately 90% of the therapeutic gain observed following OT treatment retained in follow-up (Clark et al., 2001).<br><br>Costs for the 9-month OT programme averaged $548 per subject. Post-intervention healthcare costs were lower for the OT group ($967) than for the active control group ($1,726), the passive control group ($3,334), or a combination of the control groups ($2,593). The quality of life index showed a 4.5% QALY differential (OT vs combined control), p=.001. The cost per QALY estimates for the OT group was $10,666 (95% confidence interval $6,747–$25,430). For the passive and active control groups, the corresponding costs per QALY were $13,784 and $7,820, respectively (Hay et al., 2002).	Vitality and Physical Component Score +30% decrease in the Role Physical component. No comparative or significance analyses due to the small sample size (Løkken and Overgaard, 2009).<br>The interview evaluations described that Lifestyle Redesign® gave participants, who completed, useful tools and action skills, even if living conditions worsen; knowledge of the importance of daily activities and adaptation and utilization possibilities to promote health. It resulted in changed activity execution and activity choices, increased appreciation of meaningful activities. The social benefit proved to be the driving force and main goal for the participants, and became the most important common benefit for the group (Løkken and Overgaard, 2009).
All important harms or unintended effects in each group	Nothing reported.	Nothing reported.

**Discussion**<br><br>Trial limitations, addressing sources of potential bias, imprecision, and, if relevant, multiplicity of analyses	The results may not generalize to older adults in different living situations or socioeconomic status (Clark et al., 1997; 2001), and when using differing logistical approaches to treatment administration (Clark et al., 2001). The follow-up interval is limited (Clark et al., 2001; Hay et al., 2002). Only cohort II was included in the cost-effectiveness study and attention needs to be paid to e.g. contextual factors' potential influence on the results and underlying mechanisms of effects. Since follow-up assessments were made in the absence of further treatment, the ultimate extent of cost-effectiveness/medical costs savings could not be determined (Hay et al., 2002).	No trial limitations or potential sources of bias were discussed (Løkken and Overgaard, 2009).
Generalizability (external validity, applicability) of the trial findings	The results may not be generalized to older adults in different living situations, of different socioeconomic status, or in diverse contexts (Clark et al., 1997; 2001). Limitations pertaining to generalisability of cost-effectiveness results (Hay et al., 2002).	Based on the Danish project, the authors suggested implementation in Danish municipalities, with consideration taken to participants' pre-RAND SF-36 scores, cognitive and social level of functioning, which the authors present as important success factors (Løkken and Overgaard, 2009).

Conclusion	OT, individual adaptation of program to elders' needs and contextual programme modifications, professional leadership and judgment regarded as key ingredients in enabling elders to benefit from activity (Jackson et al., 1998). Significant benefits for the OT preventive treatment group across various health, function, and quality-of-life domains. Preventive OT health programs may mitigate against the health risks of older adulthood (Clark et al., 1997), with retained benefits at long-term follow-up (Clark et al., 2001). Preventive OT demonstrated cost-effectiveness and a decreased medical expenditures trend (Hay et al., 2002).	The report authors concluded that:<br>- The Lifestyle Redesign® Programme was useful and effective for the elderly in Aalborg municipality. No statistical significance for this conclusion.<br>- Occupational therapists must be theoretically and practically schooled in the program, have access to OT professional supervision (Løkken and Overgaard, 2009); a conclusion based on the two project leaders' own experiences.
